# Monitoring functional immune responses with a cytokine release assay: ISS flight hardware design and experimental protocol for whole blood cultures executed under microgravity conditions

**DOI:** 10.3389/fphys.2023.1322852

**Published:** 2024-01-15

**Authors:** Judith-Irina Buchheim, Matthias Feuerecker, Michele Balsamo, Marco Vukich, Merel Van Walleghem, Kevin Tabury, Roel Quintens, Randy Vermeesen, Bjorn Baselet, Sarah Baatout, Bernd Rattenbacher, Inês Antunes, Thu Jennifer Ngo-Anh, Brian Crucian, Alexander Choukér

**Affiliations:** ^1^ Laboratory of Translational Research “Stress and Immunity”, Department of Anesthesiology, LMU University Hospital, LMU Munich, Munich, Germany; ^2^ Kayser Italia S.r.l, Livorno, Italy; ^3^ European Space Research and Technology Centre (ESTEC), European Space Agency (ESA), Noordwijk, Netherlands; ^4^ European Astronaut Center (EAC), European Space Agency (ESA), Cologne, Germany; ^5^ Belgian Nuclear Research Centre (SCK CEN), Radiobiology Unit, Nuclear Medical Application Institute, Mol, Belgium; ^6^ Biotechnology Space Support Center (Biotesc), Lucerne University of Applied Sciences and Arts (HSLU), Luzerne, Switzerland; ^7^ Immunology Lab, NASA Johnsons Space Center, Houston, TX, United States

**Keywords:** neuroendocrine stress response, immune monitoring, *in vitro* cytokine release assay, long-term spaceflight, functional immune testing, astronaut health

## Abstract

**Introduction:** Long-term space missions trigger a prolonged neuroendocrine stress response leading to immune system dysregulation evidenced by susceptibility to infections, viral reactivation, and skin irritations. However, due to existing technical constraints, real-time functional immune assessments are not currently available to crew inflight. The *in vitro* cytokine release assay (CRA) has been effectively employed to study the stimulated cytokine response of immune cells in whole blood albeit limited to pre- and post-flight sessions. A novel two-valve reaction tube (RT) has been developed to enable the execution of the CRA on the International Space Station (ISS).

**Methods:** In a comprehensive test campaign, we assessed the suitability of three materials (silicone, C-Flex, and PVC) for the RT design in terms of biochemical compatibility, chemical stability, and final data quality analysis. Furthermore, we thoroughly examined additional quality criteria such as safety, handling, and the frozen storage of antigens within the RTs. The validation of the proposed crew procedure was conducted during a parabolic flight campaign.

**Results:** The selected material and procedure proved to be both feasible and secure yielding consistent and dependable data outcomes. This new hardware allows for the stimulation of blood samples on board the ISS, with subsequent analysis still conducted on the ground.

**Discussion:** The resultant data promises to offer a more accurate understanding of the stress-induced neuroendocrine modulation of immunity during space travel providing valuable insights for the scientific community. Furthermore, the versatile nature of the RT suggests its potential utility as a testing platform for various other assays or sample types.

## 1 Introduction

Advancements in human space exploration, including the upcoming manned missions within the Artemis program and the development of the Lunar Orbital Platform Gateway (ESA, 2019; NASA, 2023) necessitate not only robust technical infrastructure and well-prepared crews but also the means to monitor crew health during missions. It has long been recognized that a set of stressors encountered during space flight (microgravity or µ*g*, radiation exposure, disrupted sleep, and altered circadian rhythms) subjects the body to a neurobiological stress response that profoundly affects the human immune system (Crucian et al., 2018; Buchheim et al., 2019). Although certain countermeasures, such as pre-launch quarantine protocols, have been established (Mermel, 2013), e.g., a quarantine before launch, the immune system shifts observed in returning astronauts, including viral reactivation, altered cytokine balances, and compromised T cell function, underscore the need for enhanced monitoring capabilities (Crucian et al., 2008; Crucian et al. 2014; Crucian et al. 2015). However, existing procedural and technical constraints limit the ability to perform comprehensive crew health monitoring and functional testing in space, resulting in limited insight. Immune cell counts and baseline cytokine levels in plasma are insufficient to detect and understand the distinct changes to immunity during spaceflight missions. Nevertheless, most of the functional analyses of astronauts’ bio-samples are performed on ground during pre- and post-flight time points since most of the tests require a fresh blood sample with live cells. A previously employed work-around strategy has been to download a freshly-drawn blood sample within 48 h to ground for functional testing (Crucian et al., 2015). However, no functional immune tests are currently available inflight.

In the past, inflight monitoring of crew immune health was carried out using the skin Multitest (Institut Merieux, Lyon, France), revealing compromised cell-mediated immunity in astronauts during space shuttle missions and aboard the orbital station MIR (Taylor and Janney, 1992; Gmunder et al., 1994). This test triggered a delayed-type hypersensitivity (DTH) reaction of human T cells within the skin of subjects that became visible in a timeframe of 48 h as a localized, reddened induration of which the diameter was measured. An observed reduction in size on board has led to the conclusion that cell-mediated immunity (CMI) was compromised in astronauts (Taylor and Janney, 1992; Gmunder et al., 1994). In 2002, this test was subsequently discontinued and withdrawn from the market due to antigen sensitization risks leaving a void in comparative screening options.

In response, we developed the *in-vitro* cytokine release assay (CRA), allowing for the evaluation of functional and cellular immunity, including the assessment of stress-induced alterations (Feuerecker et al., 2013). Therein, a whole blood sample is incubated *ex vivo* with a stimulus such as a viral, bacterial antigen, or mitogens for up to 48 h at +37°C to trigger an immune reaction. Thereafter, the pro and anti-inflammatory cytokines measured in the supernatant indicate the quality and quantity of the immune response. Moreover, by adding hydrocortisone to the stimulus, the neuroendocrine modulation of immune responses can be evaluated. The readout of this assay can also be performed earlier than 48 h to evaluate cell-specific activation during the phase of antigen presentation (Buchheim et al., 2018). We have previously applied this method in a clinical study of immune-compromised patients (Kaufmann et al., 2013; Feuerecker et al., 2018) and during winter-over campaigns at Concordia station in Antarctica (Feuerecker et al., 2019). Furthermore, this assay was suitable to measure the effects of simulated microgravity compared to samples incubated at 1 *g* (Van Walleghem et al., 2017) or hypergravity (Moser et al., 2023) and has been applied to study astronauts pre- and postflight after a long-term space mission to the ISS (Buchheim et al., 2019).

The interpretation of post-flight test results is on the one hand limited in capturing the intricate adaptations and alterations that occur during flight in response to the challenging environment and may on the other hand be influenced by a strong stress response during the landing phase. Thus, our concerted efforts were directed toward implementing this testing method aboard the ISS.

To accomplish this, it was necessary to devise, a novel test tube that would facilitate the handling of blood samples by astronauts in the space environment. This principle is derived from a NASA concept published in 1999, to culture whole blood during spaceflight in a µ*g* device designed to facilitate (post-culture) cells or plasma without exposing the crew to the biological sample (Crucian and Sams, 1999). The concept was further refined by NASA into a Teflon bag system to support a hypoxia investigation onboard the ISS wherein the ratio was 300 ul blood into 2.0 mL media (pre-stored in a flexible bag). This simplified concept was expanded into a test tube, with a defined 1:1 ratio of blood sample and antigen. Furthermore, the utilization of Kubik (ESA, 2018), a compact, controlled-temperature incubator, will permit the incubation of samples also under standard terrestrial gravity (1 *g*) conditions on the ISS, thereby enabling the investigation of the space environment without the impact of microgravity during the incubation period.

In collaboration with industry, we validated this test tube as flight hardware, enabling the incubation of whole blood samples with recall antigens under both µ*g* and normal gravity conditions within the Kubik incubator aboard the ISS. This process entailed defining crew procedures, rigorous material testing for biocompatibility, and the assessment of long-term frozen storage stability for stimulants. The successful integration of this hardware on the ISS in the frame of the ESA project Immunity Assay marks a significant step toward enhancing our understanding of the intricate interplay between the neuroendocrine stress response and immune functions in the challenging space environment.

## 2 Methods

### 2.1 Ethical approval

This study was approved by the local Ethical Committee of the Ludwig-Maximilians-University (LMU) Munich (protocol numbers: 269-15 and 152-06) and was carried out according to the relevant institutional and national guidelines and the World Medical Association’s Declaration of Helsinki (revised in 2013). All subjects gave their written informed consent.

### 2.2 Blood sampling

Peripheral blood samples for all sets of experiments were obtained by medically trained personnel from non-smoking, healthy donors. Blood samples were drawn into standard tubes containing lithium heparin as an anticoagulant (Monovette, Sarstedt, Germany). Filled tubes were stored at room temperature and assays were performed shortly after blood sampling. Blood sampling for end-to-end testing during a parabolic flight campaign was done during 22 s of microgravity using a peripheral venous cannula (18 G or 16 G, Braun B., Germany) which was placed on the forearm on ground before boarding the plane.

### 2.3 Principle of the whole blood cytokine release assay on ground

The CRA is executed under standard lab conditions as previously described (Feuerecker et al., 2013). In short, heparinized whole blood (500 µL) is incubated at +37°C for up to 48 h with 500 µL antigen solution containing a stimulus dissolved in Roswell Park Memorial Institute 1640 medium (RPMI; Sigma-Aldrich, Germany), or RPMI alone as a control. The stimuli and their final concentrations that were used for the CRA are bacterial recall antigen mixture (herein referred to as bacteria) containing diphtheria-, tetanus- and pertussis-toxoid (1% Boostrix^®^; GlaxoSmithKline, Germany, 10 μg/mL), Cytomegalovirus (CMV) lysate (AID - Autoimmun Diagnostika GmbH, Germany, 10 μg/mL), Epstein–Barr virus (EBV) lysate (Zepto Metrix Corp., NY, United States, 10 μg/mL), Influenza antigens (Influvac Tetra^®^, Mylan Healthcare GmbH, Germany, 10 μg/mL), *Candida* lysate (RayBiotech, Inc. Norcross, United States, 10 μg/mL), LPS (Lipopolysaccharides from *Escherichia coli* O26:B6, Sigma Aldrich, Germany, 10 ng/mL), T cell mitogens Concanavalin A (ConA, Sigma-Aldrich, Germany, 10 μg/mL) and pokeweed mitogen (PWM, Sigma-Aldrich, Germany, 10 μg/mL) or a combination of antibodies that induce direct T cell receptor activation (anti-CD3 and anti-CD28, ImmunoCult™ Human CD3/CD28 T cell activator, STEMCELL technologies, Germany, 25 μL/mL). To study the effects of immune-modulating stress hormones, hydrocortisone (Pfizer-Pharma, Germany, 0.8 μg/mL) can be added facultatively to the antigen mixture of interest for comparison of results with and without the immune-modulating capabilities of glucocorticoids. After the end of the incubation time, the supernatant generated due to gravity-induced sedimentation is collected and stored at −80°C until further analysis.

### 2.4 Reaction tube design

**FIGURE 1:**
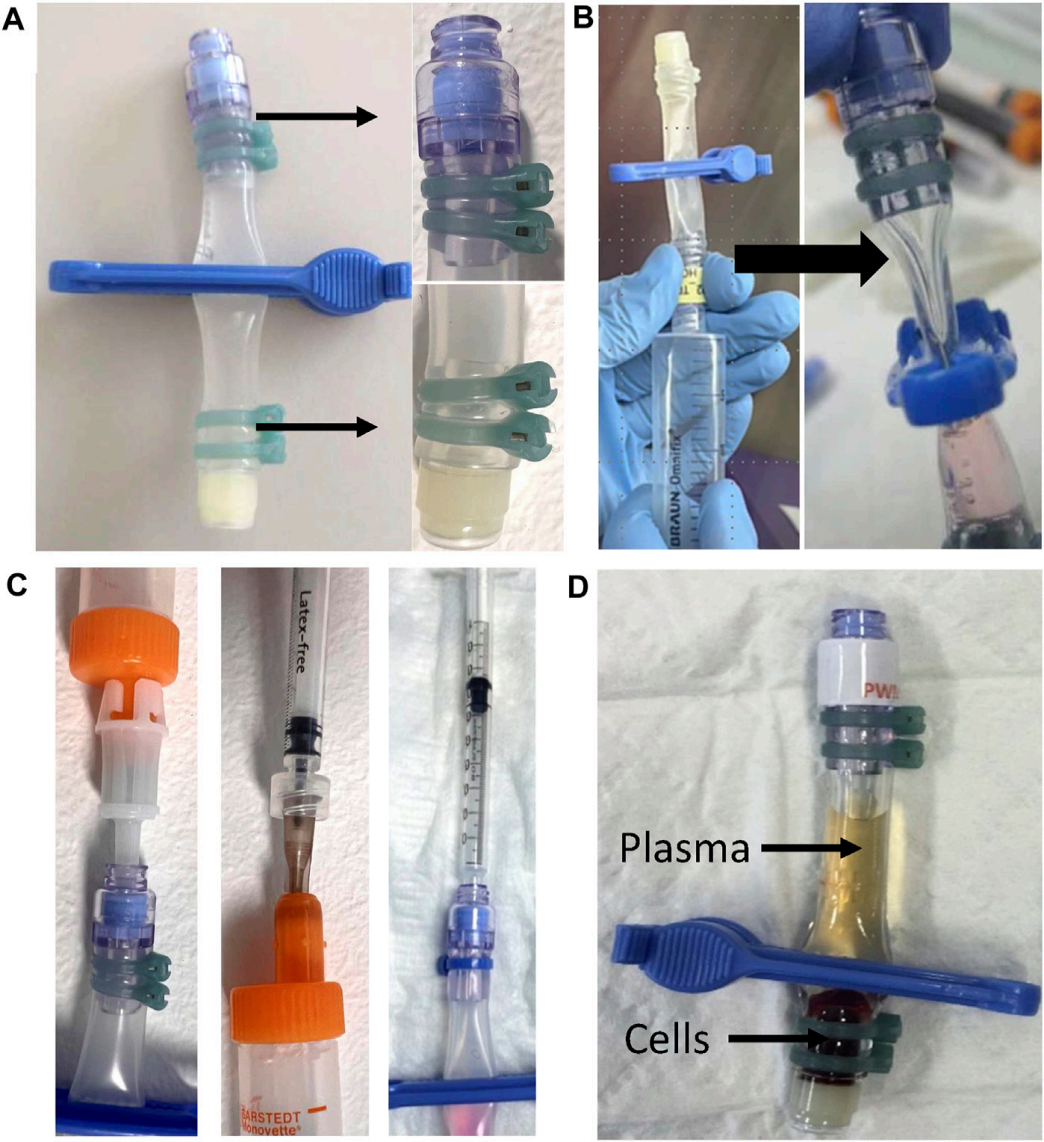
Reaction Tube design and procedural steps of the CRA **(A)** Photo showing the RT with the clip attached that is used to separate the RT into two compartments and a zoomed-in view of the secured valves. **(B)** Evacuation on the side of the swabable valve is carried out using a 20 mL syringe (left). On the right, an exemplary photo showing a clipped, prefilled RT that is sufficiently evacuated with a visibly squeezed compartment (bold arrow). **(C)** Photo showing the principle of the two tested methods for loading the blood sample into the RT after evacuation. On the left, the direct method uses the adapter, which is directly connected to the blood sampling tube. On the right, the indirect method, where first a 500 µL blood sample is aspirated into a 1 mL syringe. Then, the 1 mL syringe is pierced through the needle-free valve for a vacuum-driven filling of the compartment. **(D)** Centrifuged and clipped RT. After centrifugation, the clip is placed at the lower third of the RT to physically separate the supernatant from the cell pellet prior to frozen storage.

To harbor the CRA under microgravity conditions, a closed RT was designed and manufactured by Kayser Italia, Italy after being commissioned by ESA. The RT design consisted of a tubing and two injection valves placed at each end of the tubing. Similar to the CRA on ground, the reaction volume was planned to be 1 mL, of which 500 µL antigen mixture prefilled on Earth was to be incubated with 500 µL of whole blood sampled inflight. The maximal internal volume was calculated to be 1.10 mL with an admissible volume of 100 µL air (10% of liquid volume). As gravity-induced sedimentation is not possible under microgravity, the tube design needed to be compatible with a centrifuge and to withstand centrifugation speeds necessary to separate the supernatant from the cells. Material selection criteria were: i) sterilization possible with standard procedures; ii) biologically inert; iii) compatible with freezing at −80°C for storage; iv) sealability. Three different materials (all from Saint Gobain, France) commonly used for applications in the field of biomedical/life science were identified. Versilic-60: peroxide-cured silicone (herein referred to as silicone), C-Flex- Animal Derived Component Free Thermoplastic material (herein referred to as C-Flex), and Tygon-ND-100-65: non-di-2-ethylhexyl phthalate (DEHP) polyvinyl chloride (herein referred to as PVC), see Supplementary Figure S1A. The valves at the end facilitate the prefilling of RTs with chemicals on Earth and the loading of the sample later on board. Two types of valves, a pierceable injection valve made from polyisoprene and polycarbonate (Promepla, Principality of Monaco) and a needle-free swabable valve made from polycarbonate combined with a swabable valve stem (Qosina, United States) were evaluated and the combination of both was used in further experiments. Ty-Rap^®^ ties made of ethylene tetrafluoroethylene (ETFE, Thomas & Betts, United States) applied at each end of the tube (two per valve) were used to secure the valves and to prevent leakage (Supplementary Figures S1B, C). Separation of the reaction tube into two separate compartments to allow prefilling of RTs on ground and later the separation of supernatant and cells is necessary for the procedure. A commercial tubing clip (ATC3 tubing clip, Henley Medical Supplies, United Kingdom) was chosen for this purpose (Figure 1A).

### 2.5 RT prefilling procedure

RTs made of three different materials silicone (RT1), C-Flex (RT2), or PVC (RT3) were employed. To facilitate prefilling, the air was evacuated at the end with the injection valve using a 20 mL luer-lock syringe (Plastipak, BD, Germany) equipped with a sterile 26 G cannula [26 G × 1^1/2″^ Nr 1, Microlance™ 0.9 × 40 mm (BD, Germany) until the end of the RT appeared empty. A 1 mL syringe (BD, Germany) containing 500 µL of the desired antigen solution was attached to a new sterile 26 G cannula (BD, Germany) and used to pierce the injection valve. The volume of 500 µL was transferred to the RT driven by the previously generated vacuum. Leaving the clip attached, the RTs were then stored at −80°C until used for incubation (Supplementary Figure S1D).

### 2.6 Adapted CRA incubation procedure

Heparinized whole blood aliquots of 500 µL were transferred under aseptic conditions into the prefilled RTs driven by vacuum. First, the clipped RTs were evacuated at the side of the needle-free, swabable valve by aspiration with a 20 mL luer-lock syringe (Plastipak, BD, Germany) until visibly empty (Figure 1B). Blood samples were transferred into the RTs either directly, by connecting the standard adapter to the filled monovette and pushing it into the valve, or indirectly, by piercing the valve with a 24 G cannula attached to a 1 mL syringe (Plastipak, BD, Germany) containing 500 µL of blood (Figure 1C; Supplementary Figure S2A). Where indicated, RTs were weighed on a MP300 precision scale (Chyo, Japan) before and after prefilling as well as after the transfer of the blood sample to control the volume loaded and to detect potential deviations from the target volume. RTs were unclipped, inverted, and slightly pressed to allow mixing and incubated in a horizontal position at +37°C for 24 or 48 h. After the incubation procedure, separation of supernatant and cell pellet is done in a centrifuge. To prevent RT twisting, RTs were centrifuged in an upright position inside a 50 mL conical tube (Thermo Fischer Scientific, Germany) employing a 3D printed separator (designed by Kayser Italia, Italy) and made from Acrilonitrile-Butadiene-Stirene (ABS) + P340 (Fortus 250mc, Fortus 3D Production Systems) (Supplementary Figure S2B). After centrifugation at 600 *g* for 5 min in a centrifuge with a swing-bucket rotor (Hettich, Germany), RTs were clipped at the lower third to mechanically separate the supernatant from the cell rest. The supernatant was aspirated through the needle-free valve using a 1 mL syringe (Plastipak, BD, Germany) equipped with a 27G needle [27G × ¾^″^ Nr 20 Microlance™, 0.4 × 19 mm, (BD, Germany)] and stored in aliquots of 50 µL (Eppendorf, Germany) at −80°C until further analysis.

### 2.7 Viability assessment of cells in whole blood cultures

After the supernatant sample had been harvested, 60 µL of the cell pellet was collected and stained for viability. Samples were processed using FITC Annexin V Apoptosis Detection Kit I (BD Pharmingen, Germany) according to the manufacturer’s instructions adding a 5 min erythrocyte lysis step with lysis buffer (BD FACS lysing solution, BD Biosciences, Germany) after staining. Viability was assessed on a FACScan 9235 (Becton Dickinson Immocytometry Systems, Germany) using Cell Quest Pro software (BD Biosciences, United States). The percentages of viable (Annexin V^−^/PI^−^), early apoptotic (Annexin V^+^/PI^−^), and necrotic (Annexin V^+^/PI^+^) lymphocyte and granulocyte populations for all incubation conditions were measured.

### 2.8 Material chemical compatibility test (MCT)

Twenty-two RTs per type were disassembled (pierceable valve, swabable valve, and tubings) and all items were placed inside 15 mL closed conical tubes (Falcon, United States). The items were soaked separately in each cytokine assay mixture solution with and without hydrocortisone (total of 20 conditions) in the same concentration as planned for the experiments. While soaked, the parts also underwent thermal cycling according to the requirements of the CRA (freezing at −80°C, 72 h at +37°C, and freezing for 3 months at −80°C). All parts were then analyzed for corrosion and other damage under a stereomicroscope (Smartscope 250 MVP Automated Video Measuring Machine, OGP, United States) at ×40 magnification before and after exposure, and images were taken. To test the liquid containment of the materials after exposure to the chemicals and as such the functionality, the same items underwent a leakage test. Firstly, the items were used to reassemble the reaction tubes and, secondly, they were filled with water and weighed. Subsequently, the reaction tubes were placed in a transparent glass container to which a vacuum was applied (Pump Rz. 2.5 m from Vacuubrand, Germany). The weight reduction (water) after the test indicated a loss of containment during the test. The test and as such the type of RT was considered successful if the Leak Rate (LR) calculated after the vacuum test was less than 1 × 10^−5^ cm^3^/s applying the following formula:

$$LR=\frac{m_{loss}}{t\times 60\times \rho }\times \frac{{DP}_{1}}{{DP}_{Test}}$$

where: *LR* = leak rate, [cm^3^/s], *m*_*loss*_ = water mass loss, [g], measured with scale accuracy of 0.001 g, t = testing time, [min.] equal to 60 min, ρ = assay mixture density, [g/cm^3^], equal to 1 g/cm^3^, *DP*_1_ = [bar], equal to 1 bar, *DP*_*TEST*_ = Test Delta Pressure, equal to 1 bar.

### 2.9 Parabolic flight campaign and simulation of crew procedure

The objective of the parabolic flight experiment was to perform end-to-end testing of critical operational steps such as blood sampling, the air bubble-free sample transfer into tubes, mixing of samples with tube contents, centrifugation, and clipping of the RT under μ conditions. Twenty RTs of all three types were prefilled with RPMI on ground according to the standard procedure. All key procedural steps were tested under µ*g* conditions during a parabolic flight campaign (VP119) at Novespace (Bordeaux, France). On ground, an intravenous 18 G cannula (B. Braun, Germany) was placed on both forearms of each donor and secured with tape and bandages. Blood sampling under µ*g* was done by placing the arm in a sealed plastic bag (Toppits^®^, Germany) to prevent any blood from spilling inside the compartment of the aircraft. Four standard sizes of sampling tubes employed with a plunger and containing lithium heparin as an anticoagulant (all from Sarstedt, Germany) were tested: 2.7, 4.5, 7.5, and 9 mL. The operator filled the desired blood tube within the 22 s of µ*g* during the flown parabola by gently pulling the plunger. Next, the filled blood sampling tubes were subjected to gentle movements (20 s floating, impulses like pushing but not spinning) to mimic a transfer of the tube to the area on the ISS where the next procedural steps would take place. The location of the air inside the monovette was video documented. Further steps continued inside a polycarbonate glove box (∼80 L) to work with the blood samples in a liquid-tight environment. Blood samples were transferred into previously evacuated RTs using the two described transfer methods, either directly (via standard adapter) or indirectly (via cannula and 1 mL syringes). Adequate filling of RTs was evaluated by photo documentation and by determination of the mass using an MP300 precision scale (Chyo, Japan). We evaluated placing the clip in microgravity on 5 RTs per RT type. This step is necessary to ensure the permanent separation of the supernatant from the cellular rest during storage. RTs were placed in a swing-bucket rotor centrifuge (Eppendorf, Germany) inside a separate aluminum box (Zarges, Germany, 260 L) and the centrifugation step (1,500 *g* for 5 min) was planned such that the centrifuge was started under 1 *g* and stopped when the 22 s phase of µ*g* had begun. Tubes were centrifuged inside plastic bags in case of inadvertent spilling of blood. Once the centrifuge stopped, the RTs were lifted out of the holder and clipped during µ*g*. The quality of separation was documented.

### 2.10 Test of interfaces between RTs and ISS incubator Kubik

The test procedure was carried out at the Biotechnology Space Support Center (BIOTESC), University of Luzerne (USLU), Switzerland using the accessible ground model of the Kubik incubator (FM2, ESA). A previously used (Hatton et al., 1998) Kayser Italia Container-Spider (KIC-Spider, Kayser Italia, Italy) was combined with a 3D printed insert (Kayser Italia, Italy) and evaluated for insertion of RTs in Kubik. This KIC insert (length 40.30 mm, width 20.30 mm, height 85.10 mm, and mass 10.90 g) was manufactured in Acrilonitrile-Butadiene-Stirene (ABS) + P340 through 3D printing (Fortus 250mc, Fortus 3D Production Systems) and contains 4 bays for harboring 4 RTs at a time. We also evaluated a short centrifugation step of up to 2 min to improve the mixing of the two compartments of blood sample and antigen solution under µ*g*. Pass criteria for the centrifugation step were: i) the liquid is collected at the bottom of the tube and ii) major air bubbles could be removed from the liquid after centrifugation.

### 2.11 Material biocompatibility test (MBT)

For the MBT, the standard control tube (flat bottom 1.5 mL cryotube, Nunc, United States) and a total of 32 per RT type were filled with either bacteria, PWM, CD3/CD28, or RPMI (control) and incubated at +37°C for 24 h and 48 h with whole blood samples, which were drawn from 4 individual donors. The amount of interleukin (IL)—2, IL-6, IL-10, tumor necrose factor (TNF), and interferon γ (IFNγ) was measured in the supernatant after 24 and 48 h. Data from all types of RTs were compared to the laboratory standard.

The following success criteria were applied: The MBT of the distinct RTs was considered successful when i) control samples differed only in the range of 10% from results obtained with the RT; ii) comprised a maximum of 10% necrotic cells after incubation; and iii) a maximum of 50% (25% for lymphocytes, 50% for granulocytes) of early-stage apoptotic cells after incubation.

### 2.12 Short and long-term storage of antigen stimuli inside RTs: stability testing campaign

A total of 80 RT2 and 80 RT3 were filled at the same time each with one of 8 different types of stimuli (Bacteria, *Candida*, CD3/CD28, EBV, LPS, PWM, PWM with hydrocortisone, and RPMI as control) and subsequently frozen at −80°C. After 2 days (T_0_), then 2 months (T_1_), and 3 months (T_2_) 16 RT2 and 16 RT3 were thawed. Each stimulus was run in duplicates for the CRA as described using blood samples drawn at the same time from a single donor. At each time point, all 32 RTs were run on the same date, and RT mass was logged. Following the completion of the CRA, the clipped RTs were subjected to freezing to simulate conditions comparable to those aboard the ISS thereby replicating the same number of freeze and thaw cycles. Shortly after, the RTs were thawed, and the supernatant was analyzed to ascertain the viability of achieving adequate stimulation even after prolonged storage periods in the freezer. The selection of a specific material necessitated a decision within 3 months of storage to ensure timely implementation on the ISS. Subsequent evaluations to assess storage effects were conducted similarly at 8 (T_3_) and 9 (T_4_) months after the freezing of the antigens in the RTs, but only with the remaining RT type.

### 2.13 Detection of cytokines

The supernatants were analyzed for cytokines (Il-2, IL6, IL10, TNF, IFNγ) during Biocompatibility Testing using the BD™ Cytometric Bead Array Human Th1/Th2 Cytokine Kit II (CBA, BD Biosciences, Germany) on a FACScan9235 device (Becton Dickinson Immocytometry Systems, Germany). For the Stability Test of the Antigens, the MILLIPLEX^®^ Human Cytokine/Chemokine/Growth Factor Panel A—Immunology Multiplex Assay, Merck, Germany was used on a MagPix device (Luminex Corporation, Austin, Texas) according to the manufacturer’s instructions. Results are provided as mean fluorescence intensity (MFI) or concentration (pg/mL) where indicated.

### 2.14 Reevaluation of centrifugation speeds

RTs were evaluated for leakage during centrifugation and leakage, if observed, was documented. A simplified test run was done with 16 RT2 and 16 RT3 during the stability testing at timepoint T_2_ where different centrifugation speeds (600–1,000 *g*) were tested.

### 2.15 Statistical analysis

Statistical evaluation and plotting of the data set were done using SigmaPlot 13 (Systat Software Inc., United States). Data are described as mean ± standard deviation (SD). MFI values derived from CRAs during the biocompatibility testing are plotted as single-subject data on a log scale. After testing for normality using the Shapiro-Wilk Test, data was evaluated with repeated measures of one-way repeated measures (RM)-ANOVA followed by the Holm-Sidak *post hoc* test for multiple comparisons. We compared storage conditions with the standard lab procedure of the CRA as well as long-term storage up to 9 months. A value of *p* < 0.05 was considered statistically significant.

## 3 Results

### 3.1 Reaction tube handling on ground

Handling of the reaction tube design with both a pierceable and a needle-free valve fixed with ties at each end made from either Silicone (RT1), C-Flex (RT2), or PVC (RT3) was tested. For all three materials, evacuation and prefilling were achieved without problems. Anomalies such as clotting of blood samples were never observed. The vacuum-driven transfer of 500 µL of whole blood from the sampling tube into the RTs using the direct method with the adapter attached to the needle-free valve was found both feasible and easy to perform (Supplementary Figure S2A). After incubation, the centrifugation of RTs inside the conical tube using the designed separator prevented twisting of the RTs and allowed a clear separation of supernatant and cells with all materials (Supplementary Figures S2B, C). Clipping of the tube using the commercial clip was possible without any difficulty and did not compromise the materials in any way (Figure 1D).

### 3.2 Material chemical compatibility test (MCT)

Inspection of components showed no weakness of materials independent of the kind of tubing or substance used (data not shown). To functionally test liquid containment after exposure to the chemicals, the reassembled reaction tubes were filled with water and underwent a leakage test in a vacuum chamber. All RT designs evaluated in the test showed no leakage and thus the test was considered successful for all RT types (Supplementary Table S1).

### 3.3 Material biocompatibility test (MBT)

**FIGURE 2:**
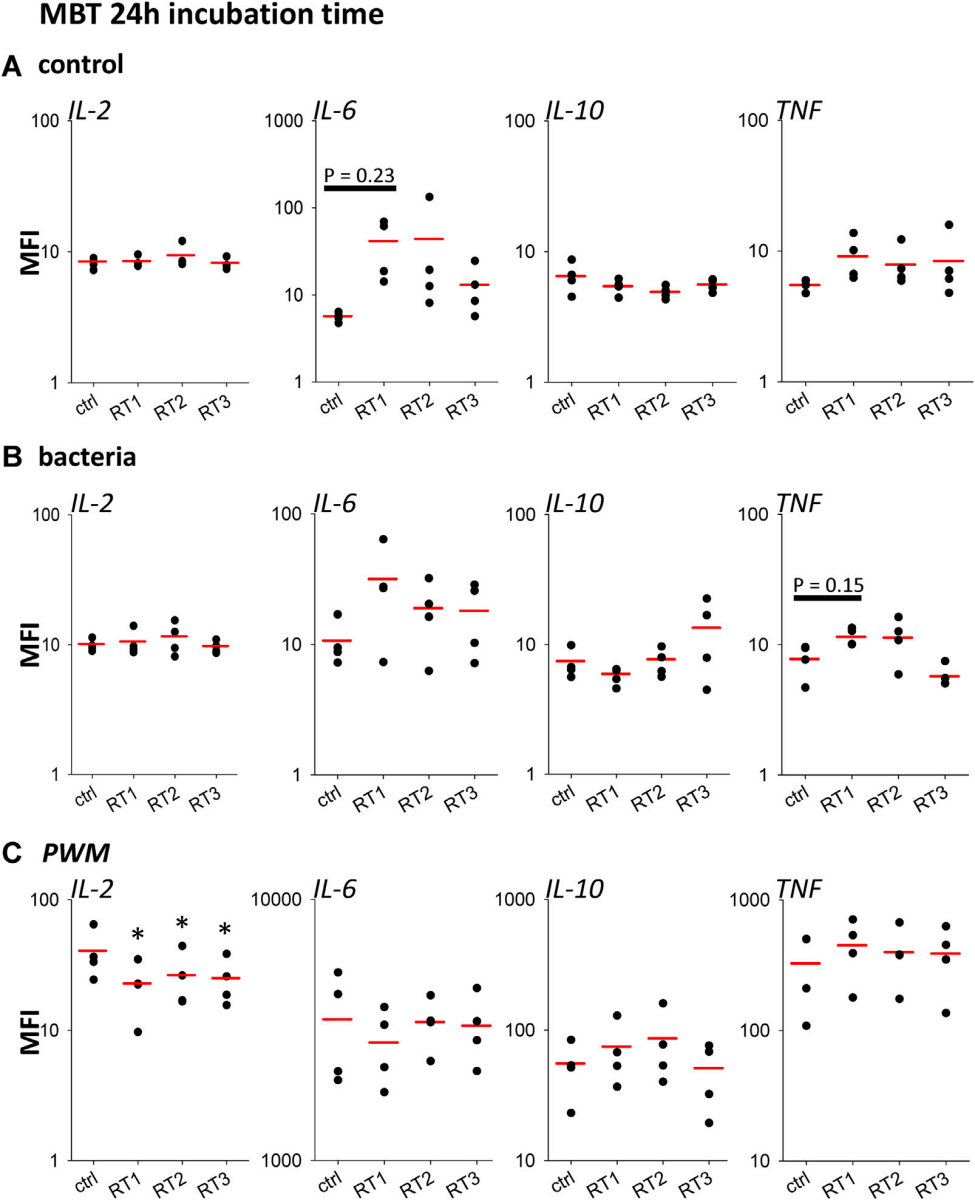
Biocompatibility of materials 24 h. Scatter plots showing amounts of IL-2, IL-6, IL-10, and TNF detected in supernatants after incubation of blood samples for 24 h with RPMI (Control), **(A)**, bacteria **(B)** and PWM **(C)**. The three types of RT Silicone (RT1), C-Flex (RT2), and PVC (RT3) were compared to the established standard setup (ctrl). The red line represents the mean, *n* = 4 individual subjects, *p < 0.05 vs. ctrl.

The amount of cytokines IL-2, IL-6, IL-10, TNF, and IFNγ was measured in the supernatant after 24 h (Figure 2) and 48 h (Supplementary Figure S3) without stimulation (control) or after incubation with either bacteria, PWM, or CD3/CD28. Data from all types of RTs were compared to the laboratory standard. Under control conditions, we mostly observed similar results in all types of reaction tubes compared to the laboratory standard. Significant differences compared to the standard tube were seen for all RT types with PWM and IL-2 at 24 and 48 h and for RT1 only for IL-6 after incubation with bacteria and PWM at 48 h. However, a similar trend was seen already at 24 h which was not statistically significant.

### 3.4 Viability assessment for MBT

The percentages of apoptotic and necrotic cells were assessed after 24 h and 48 h of incubation with all recall antigens in the 3 different reaction tubes and control tubes to determine the influence of the materials and experimental set-up on the viability of cells. Notably, a significant increase in the fraction of necrotic lymphocytes was observed in all tested RTs after 24 h incubation of whole blood cultures with CD3/CD28 compared to control tubes, which was not observed after 48 h. Overall, a low mean percentage (<2% after 24 h and 48 h) of necrotic or apoptotic lymphocytes was detected in all RTs for all stimuli and no significant differences between the different RTs were detected (Table 1).

**TABLE 1:** Viability test.

Lymphocytes 24 h	Standard tube		RT1		RT2		RT3	
	Apoptotic	Necrotic	Apoptotic	Necrotic	Apoptotic	Necrotic	Apoptotic	Necrotic
Control	1.28 ± 0.46	0.13 ± 0.21	1.16 ± 0.38	0.04 ± 0.02	0.84 ± 0.21	0.01 ± 0.01	1.60 ± 0.53	0.01 ± 0.01
Bacteria	0.85 ± 0.26	0.02 ± 0.01	1.59 ± 0.84	0.02 ± 0.01	1.14 ± 0.76	0.03 ± 0.02	1.38 ± 0.66	0.04 ± 0.01
PWM	0.64 ± 0.21	0.05 ± 0.03	1.31 ± 0.73	0.10 ± 0.03	1.21 ± 0.26	0.54 ± 0.07	1.26 ± 0.25	0.85 ± 0.41*
CD3/CD28	0.80 ± 0.26	0.06 ± 0.02	0.87 ± 0.27	0.38 ± 0.23*	1.05 ± 0.47	1.25 ± 0.37*	0.79 ± 0.12	0.76 ± 0.28*

Lymphocytes 48 h	Standard tube		RT1		RT2		RT3	
	Apoptotic	Necrotic	Apoptotic	Necrotic	Apoptotic	Necrotic	Apoptotic	Necrotic
Control	1.12 ± 0.33	0.08 ± 0.10	1.57 ± 0.94	0.02 ± 0.02	1.04 ± 0.24	0.04 ± 0.02	1.13 ± 0.27	0.02 ± 0.01
Bacteria	1.10 ± 0.37	0.21 ± 0.26	1.29 ± 0.35	0.02 ± 0.02	1.41 ± 0.42	0.04 ± 0.01	1.37 ± 0.20	0.33 ± 0.48
PWM	0.87 ± 0.17	0.34 ± 0.21	1.44 ± 0.41	0.64 ± 0.85	1.02 ± 0.35	0.64 ± 0.26	1.14 ± 0.11	0.86 ± 0.29
CD3/CD28	0.48 ± 0.33	3.01 ± 2.50	0.90 ± 0.36	0.84 ± 0.11	1.13 ± 0.42	1.19 ± 0.57	0.70 ± 0.15	1.03 ± 0.69

Percentage of early apoptotic and necrotic lymphocytes after incubation of whole blood cultures with stimuli for 24 and 48 h in control tubes or RTs. Unstimulated samples served as controls. Data (*n* = 4) is listed as mean ± SD., Significant differences compared to the respective control tube results are indicated by *p < 0.05 (Repeated measures one-way ANOVA, with Holm-Sidak *post hoc* test).

### 3.5 Deselection of one material after MBT

As a result of the MBT, one less favorable material had to be deselected to proceed. Although all three materials performed well, and neither the MCT nor viability tests identified a major outlier, we deselected silicone/RT1 from future tests. The rationale was in part because we noticed slightly more significant deviations from the rest of the RTs and also in part because of the handling. Silicone was very soft and flexible, whereas the filling procedure benefited from a slightly more rigid tube.

### 3.6 Testing of critical procedural steps in a parabolic flight campaign

**FIGURE 3:**
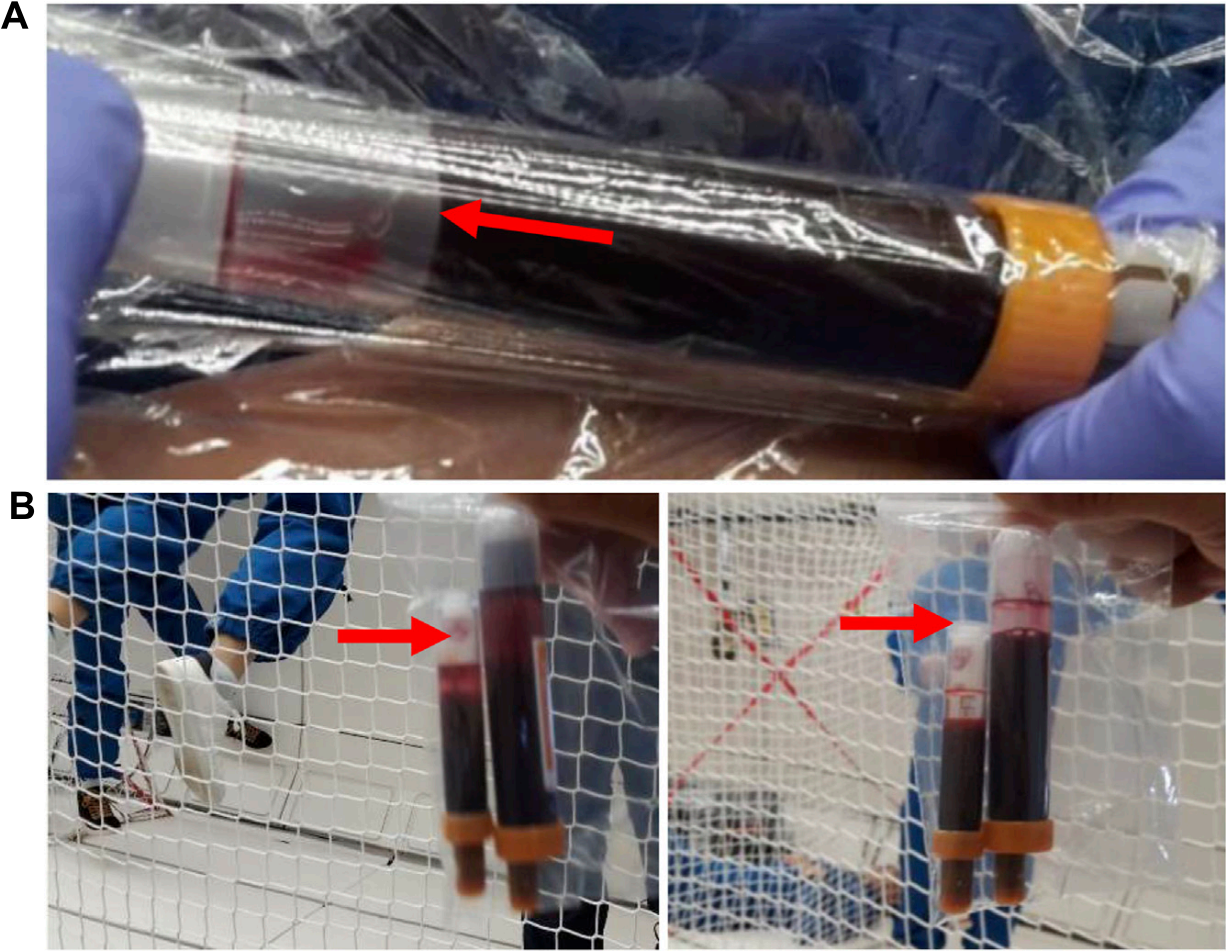
Parabolic flight campaign. Exemplary photos showing distinct steps of the CRA procedure during parabolic flight. **(A)** Setup of the blood draw under microgravity conditions. Plastic wrap was necessary to prevent inadvertent blood spilling. 9 mL Blood sampling tube at the time of being filled during the 22s of µ*g*. The air pocket (red arrow) remains at the end of the tube (left side) when the plunger is gently pulled. **(B)** Photos showing blood sampling tubes that have been moved gently during microgravity (left side) and shortly after (right side). The air pocket (red arrow) remained located at the end of the tube.

Mean delta mass and SD were within the expected parameters during prefilling on ground (RT2/C-Flex: 0.521 ± 0.02 g, and RT3/PVC: 0.513 ± 0.01 g, *p* = 0.147). The execution of blood sampling under µ*g* showed that by a gentle continuous aspiration of the monovette’s plunger, the air remained at the end of the tube close to the plunger as a large single air bubble (Figure 3A). Air and blood remained separate independent of tube size. Moving the tube around in the plane did not result in the mixing of the two phases of blood and air. This effect was successfully tested for the 9 mL and 2.7 mL tubes (Figure 3B). Evacuation of the RTs was feasible in μ*g* if performed shortly before filling of the RTs and the direct transfer method was both feasible and preferable to the indirect method for the operator. Upon using a 1 mL syringe to transfer the sample into the RT, it became clear that the aspiration process resulted in many air bubbles inside the syringe. This often led to air being transferred into the RT, Furthermore, handling the needle is a potential safety hazard. The direct method, which avoids handling the needle, was therefore chosen. Interestingly, some of the adapters became clogged when used multiple times. We therefore continued to use each adapter only once. Filling of RT2 (Supplementary Video S1) and RT3 (Supplementary Video S2) occurred within seconds. After unclipping, RTs continued to be notched in the middle, which impeded mixing. This hourglass shape was most pronounced in the rigid RT3/PVC tube. Pressing on the notch opened the tube and allowed mixing but the RT still appeared slightly compressed in the middle. Irrespective of tube material mixing (squeezing and shaking) in μ*g* resulted in many air bubbles distributed all over the RT. These air bubbles quite constantly remained in place, similar to the large air bubble inside the blood sampling tube. Squeezing and shaking were both necessary to enable the mixing of the two components under 1 *g*. Furthermore, a short *g*-impact (hypergravity phase of the parabola: 1.8 *g* for 22 s) was sufficient to bring the liquids back to one level and only small air bubbles remained close to the blue valve. It was therefore concluded to subject all tubes to a very short 1–2 *g* centrifugation inside Kubik before the actual incubation period to improve the mixing of the antigen and the blood sample inside the RT.

On the ISS, RTs would then undergo incubation at 1 *g* or 0 *g* inside Kubik. Kubik insertion was not tested during this campaign. After incubation and removal from Kubik all RTs need to undergo a centrifugation step to separate the supernatant from the pellet prior to placing the clip. Centrifugation at 1,500 *g* for 2–5 min resulted in a clear separation of cell pellet and supernatant. Taking the samples out of the centrifuge, which was optimized to stop during the μ*g*-phase, showed repeatedly that this procedure does not lead to the re-mixing of the two separated phases. Clipping was repeatedly successful in µ*g* for all tested RTs at the area of the tube that is midway between the level of blood and the notch (lower third of the RT) seemed an optimal reference to avoid remixing and to achieve the desired volume of supernatant. In case of re-mixing, another centrifugation cycle can be performed.

Mean delta mass and SD were established for those RTs where visual control showed good filling. These observations were confirmed by the mass log (RT2/C-Flex: 0.514 ± 0.03 g and mean total mass 1.034 ± 0.04 g, and RT3/PVC: 0.477 ± 0.04 g and mean total mass 0.994 ± 0.03 g; *p* = 0.002)

The following recommendations and lessons learned were reported:

1. Careful blood sampling (soft pulling at the plunger) and handling in μ*g* will result in a blood-filled monovette showing one single air pocket at the region of the plunger.
2. The blood transfer shall be done directly with the multifly adapter connected to the monovette. For each RT, a new multifly adapter shall be used.
3. To facilitate the mixing of blood sample and antigen solution after unclipping, all RTs shall undergo a short spin (1–2 min) at 1–2 *g* inside Kubik.
4. Centrifugation at 1,500 *g* for 5 min resulted in a clear separation and clipping of tubes shall be performed at the lower third of the tube.

### 3.7 Evaluation of effects for long-term storage

**FIGURE 4:**
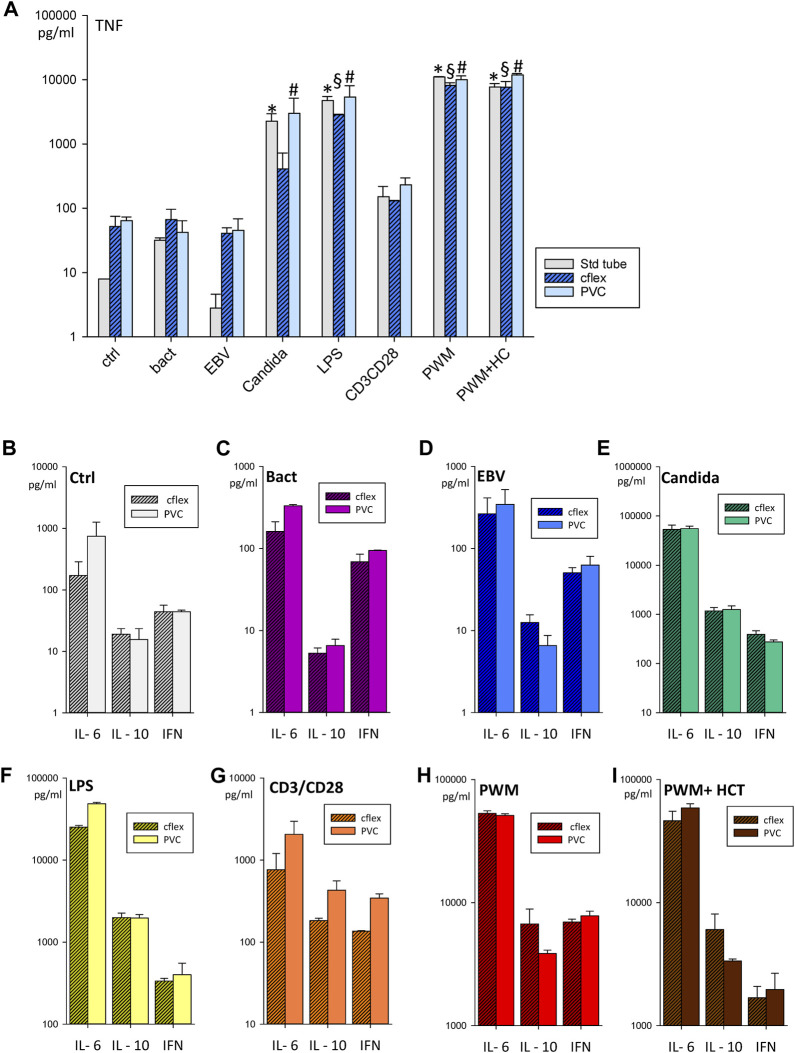
Stability testing of antigens **(A)** Bar chart showing TNF concentrations in supernatants after incubation for 24 h with bacteria, EBV, *Candida*, LPS, CD3/CD28, PWM, PWM with hydrocortisone, and RPMI as control (ctrl). Data were generated in the standard tube, C-Flex/RT2 and PVC/RT3 at the time point T_0_ of the stability testing campaign. Bar charts showing cytokine concentrations of IL6, TNF, IFN, and IL-10 in a direct comparison of C-Flex/RT2 and PVC/RT3 for the control condition **(B)**, bacteria **(C)**, EBV **(D)**, *Candida* **(E)**, LPS **(F)**, CD3/CD28 **(G)**, PWM **(H)**, and PWM with hydrocortisone **(I)**. Data are expressed as mean ± SD, * = *p* < 0.05 vs. the standard tube ctrl, § = *p* < 0.05 vs. the C-Flex ctrl. # = *p* < 0.05 vs. the PVC ctrl. Results were generated from duplicates generated by a single blood draw of one donor.

**FIGURE 5:**
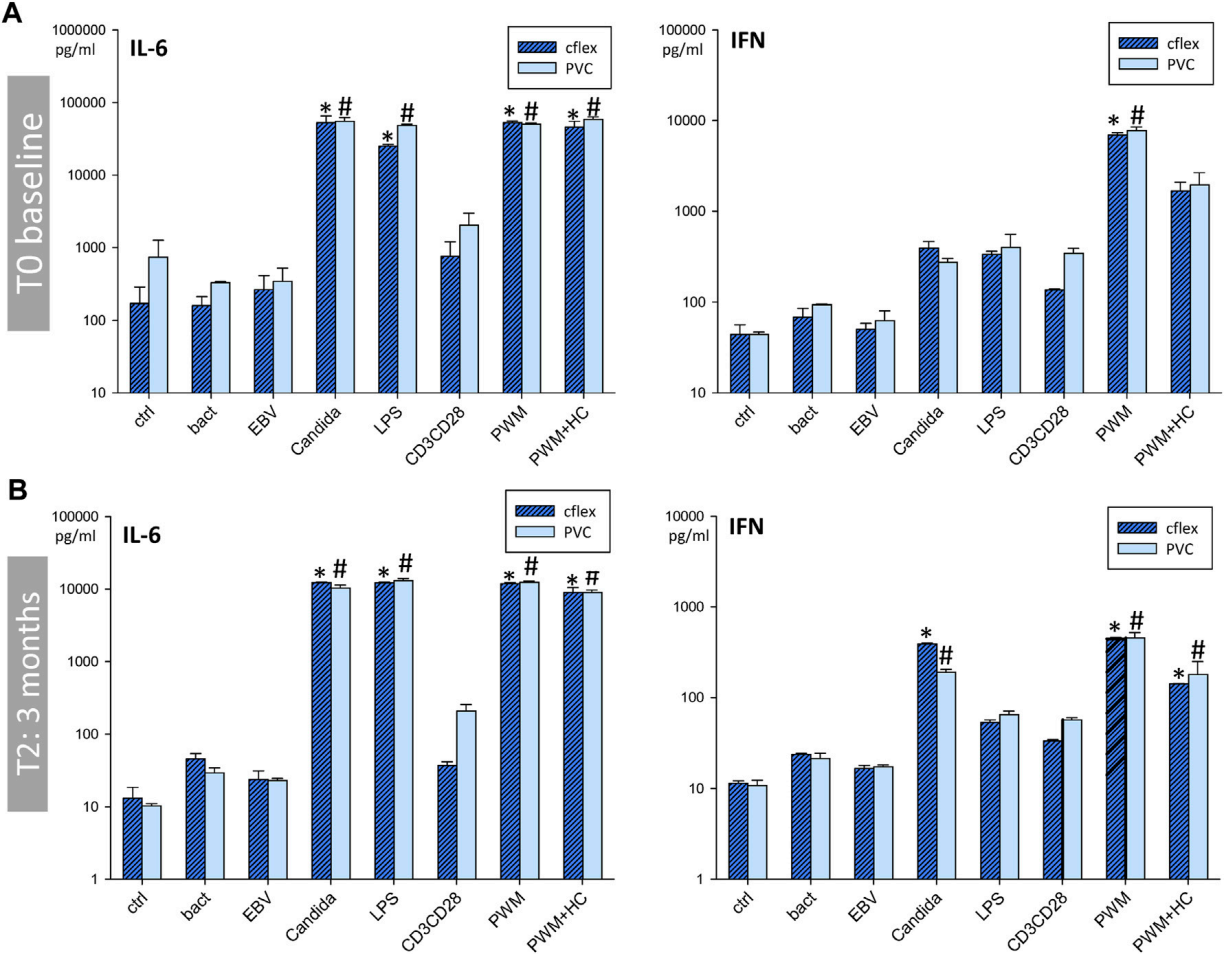
Comparison of C-Flex and PVC during stability testing: T_0_ andT_2_. Bar charts showing IL-6 and IFN concentrations in supernatants after incubation in C-Flex/RT2 or PVC/RT3 for 24 h with bacteria, EBV, *Candida*, LPS, CD3/CD28, PWM, PWM with hydrocortisone, or RPMI as control (ctrl). Data are shown for the timepoint T_0_ baseline **(A)**, and T_2_ 3 months **(B)** after baseline and are expressed as mean ± SD* = *p* < 0.05 vs. C-Flex ctrl., # = *p* < 0.05 vs. PVC ctrl. Results were generated duplicates generated at each time point by a single blood draw of one donor.

**FIGURE 6:**
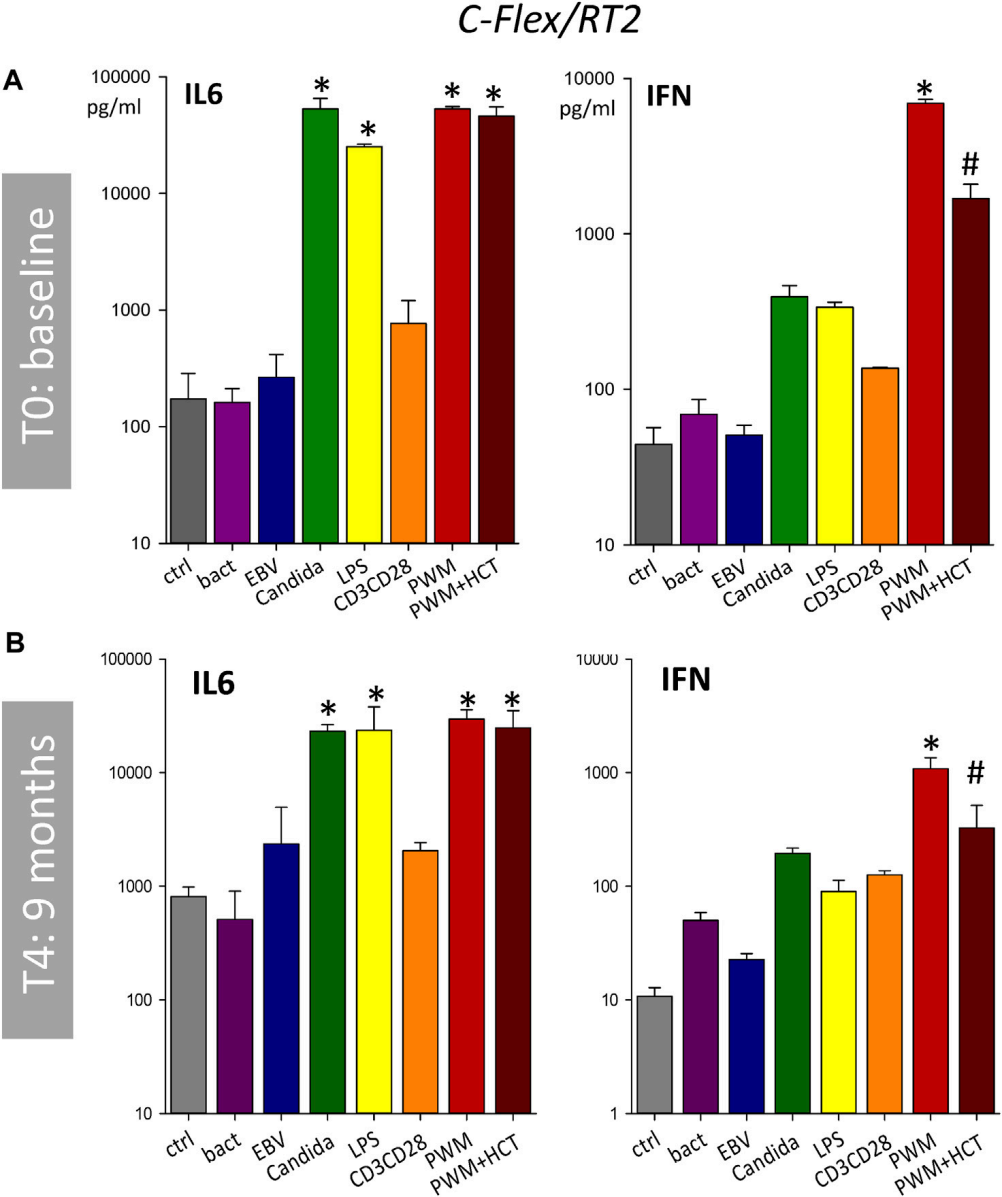
Long-term storage of antigens in C-Flex/RT2. Bar charts showing IL-6 and IFN concentrations in supernatants after incubation in C-Flex/RT2 for 24 h with bacteria, EBV, *Candida*, LPS, CD3/CD28, PWM, PWM with hydrocortisone, or RPMI as control (ctrl). Data are shown for the timepoint T_0_ baseline **(A)**, and T_4_ 9 months **(B)** after baseline and are expressed as mean ± SD, * = *p* < 0.05 vs. ctrl. # = *p* < 0.05 PWM vs. PWM + HCT. Results were generated from duplicates generated by a single blood draw of one donor.

Since it became evident, that a 24 h incubation time would be preferable for on-board operations, we focused in the next analyses on the 24 h timepoint. At T_0_ both RTs showed a similar immune response profile compared to the established standard tube (Figure 4A). A direct comparison between C-Flex/RT2 and PVC/RT3 (Figures 4B–I) evidenced no significant differences between RT types or antigen responses. At the following time points 2 months (T_1_, data not shown) and 3 months (T_2_) after storage began, similar results were seen for the cytokines IL-6, IFNγ (Figure 5) or TNF and IL-10 (data not shown.) Antigen activation worked fine in both tube types and similar profiles were observed. Interestingly, suppression of an immune response by hydrocortisone during stimulation with PWM was only seen for IFNγ release. Leakage of blood out of the RT during the centrifugation step was observed at higher speeds (>1,000 *g*) mostly for PVC tubes (Table 2). Reducing the speed to 600 *g* prevented leakage in PVC/RT3. Since implementation was imminent, we had to focus our efforts on one material, and C-Flex/RT2 was chosen due to the slightly better handling quality and also due to a much smaller risk for leakage. The long storage time points T_3_ (data not shown) and T_4_ were therefore only executed with C-Flex/RT2. Similar to the previous finding a typical immune profile was detected with significant alterations of cytokines IL-6, INFγ (Figure 6) as well as IL-2, TNF, or IL-10 (data not shown). Interestingly, we observed higher IL-6 levels at 9 months (T_4_) in this single subject after incubation with bacteria, EBV, or control. When asked, the subject reported a very recent respiratory viral infection that the person was recovering from.

**TABLE 2:** Leakage evaluation of RT2 and RT3 at distinct centrifugation speeds.

	Condition	Material	Speed (g)	Time (min)	Leakage (Y/N)	Separation (Y/N)
1	ctrl	C-Flex/RT2	1,000	5	**N**	Y
		PVC/RT3			**N**	Y
	Bacteria	C-Flex/RT2			**N**	Y
		PVC/RT3			**Y**	Y
2	ctrl	C-Flex/RT2	900	5	**N**	Y
		PVC/RT3			**N**	Y
	Bacteria	C-Flex/RT2			**N**	Y
		PVC/RT3			**N**	Y
3	EBV	C-Flex/RT2	800	5	**N**	Y
		PVC/RT3			**Y**	Y
	*Candida*	C-Flex/RT2			**N**	Y
		PVC/RT3			**N**	Y
4	EBV	C-Flex/RT2	700	5	**N**	Y
		PVC/RT3			**N**	Y
	*Candida*	C-Flex/RT2			**N**	Y
		PVC/RT3			**N**	Y
5	CD3/CD28	C-Flex/RT2	700	5	**N**	Y
		PVC/RT3			**Y**	Y
	LPS	C-Flex/RT2			**N**	Y
		PVC/RT3			**N**	Y
6	CD3/CD28	C-Flex/RT2	700	5	**N**	Y
		PVC/RT3			**Y**	Y
	LPS	C-Flex/RT2			**N**	Y
		PVC/RT3			**N**	Y
7	PWM	C-Flex/RT2	600	5	**N**	Y
		PVC/RT3			**N**	Y
	PWM + HCT	C-Flex/RT2			**N**	Y
		PVC/RT3			**N**	Y
8	PWM	C-Flex/RT2	600	5	**N**	Y
		PVC/RT3			**N**	Y
	PWM + HCT	C-Flex/RT2			**N**	Y
		PVC/RT3			**N**	Y

Table summarizes the observations of leakage when subjecting RT2 and RT3 to different centrifugation speeds ranging from 600 *g* to 1,000 *g*. Leakage was observed only in some of the PVC, tubes (red font). N = No, observation not made, Y = Yes, observation made.

### 3.8 Kubik interface test and pre-incubation downspin

**FIGURE 7:**
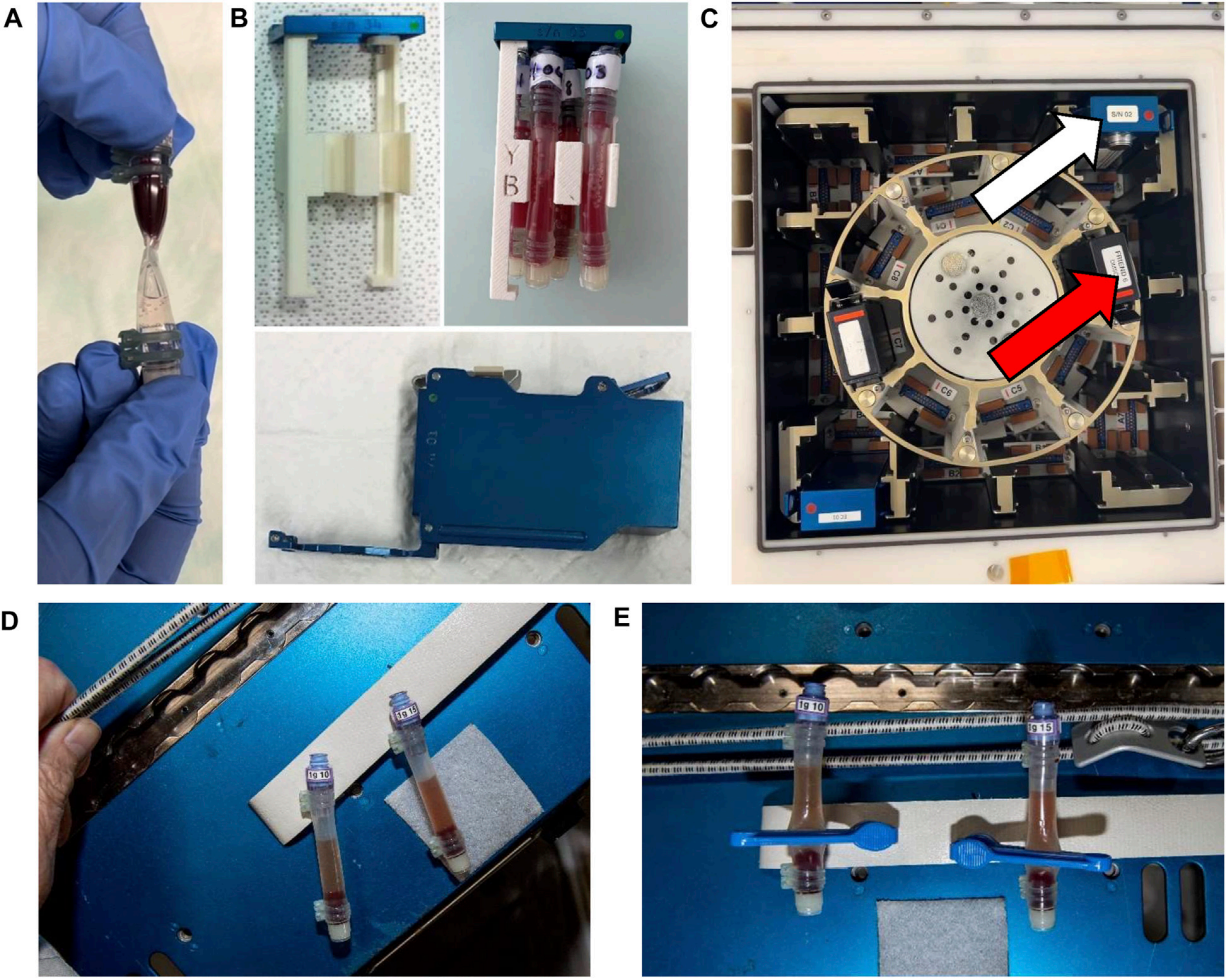
Evaluation of the interface of RTs with the Kubik ground model **(A)** Photo showing the hourglass shape, typical for RTs after unclipping, which can interfere with the mixing of antigens and sample. **(B)** KIC (bottom) and KIC insert (up left). The KIC insert harboring 4 RTs (2 PVC and 2 C-Flex) supporting the round shape of the RT during the incubation time. **(C)** KIC inserted in a centrifuge’s slot (red arrow), KIC inserted in a static position’s slot (white arrow). **(D, E)** First images of the hardware after being used for the first time for the CRA on board the ISS in the frame of the ESA project IMMUNITY ASSAY (Courtesy of ESA/NASA). Figure D shows Two RTs after 24 h incubation in Kubik and after centrifugation at 600 *g* for 5 min. Cell pellet and supernatant are visibly separated. Figure E shows two tubes after clipping just before frozen storage. The clips were successfully placed to fix the separation of supernatant and cell pellet before freezing.

We evaluated the fit of the 3D-printed KIC insert for Kubik and the efficiency of mixing blood and antigens by applying short downspin steps at low *g*. After filling the RTs (RT2 and RT3) with antigen and blood samples as per procedure, the clip was removed from the RTs. The center part where the clip divides the tubes into two compartments was gently squeezed and thus opened to allow the liquids to mix (Figure 7A). While squeezing, air bubbles were visible inside the liquids. Two PVC and two C-Flex tubes were inserted into the KIC insert. Care was taken to completely push the tubes toward the blue lid and a good fit was achieved. The rigid frame of the KIC insert forced the RT types back to the favorable cylindrical shape and thus facilitated direct mixing (Figure 7B). RT2/C-Flex was easier to handle since it was less rigid than RT3/PVC. On orbit, the centrifugal force provided by Kubik will collect the liquid on the side wall. On ground, if Kubik remains in an upright position, the blood will flow to the bottom of the tube as soon as the centrifugation is stopped. After centrifugation at 2 *g* for 1 or 2 min, respectively, the lid was opened and the KICs were removed without tilting Kubik nor the KIC (Figure 7C). After 1 min of centrifugation, bubbles could still be seen in the liquid and the mixing did not appear to be good whereas after 2 minutes the bubbles were gone from the liquid (Table 3). The 1-min operation was therefore discarded. To test the quality of mixing, the tubes were reclipped in the middle and the liquid was removed from both sides separately and transferred into 1.5 mL Eppendorf tubes. The tubes were centrifuged at 2,500 *g* for 5 min (Eppendorf, Germany) at room temperature. The volume of the supernatant V(S) as well as the volume of the pellet V(P) that contains the blood cells were measured. It was expected that samples if properly mixed would give the same ratio V(S)/V(P) on both sides. After 2 min of centrifugation, the V(S)/V(P) ratio was not satisfactory, yet. Therefore, a third test was performed exactly as the other tests but after unclipping, the contents of both sides were mixed by gently squeezing the tube alternating between the yellow and the blue side. Although the ratios V(S)/V(P) were not equal it seems the mixing in this case was slightly better than in the other cases (Table 4). The KIC and its insert were found easy to handle and interfaced without problems. To facilitate mixing in µ*g*, the RTs had to be squeezed gently at the area of the clip to open the crimped middle part. Before incubation, all RTs need to be subjected to short centrifugation in Kubik for 2 minutes at 2 *g*, which will expel air bubbles from the liquid and improve mixing. On orbit, liquids of RTs in the 1 *g* compartment will collect on the sidewall of the RT directed outwards- On the ground, this is mimicked by placing RTs in a horizontal position. The two different tubes, C-Flex and PVC both formally passed the test, whereas the C-Flex tubes were found easier to handle compared to the PVC.

**TABLE 3:** Kubik centrifugation test.

	1 min		2 min		2 min and squeezing	
	C-Flex	PVC	C-Flex	PVC	C-Flex	PVC
Leakage	No	No	No	No	No	No
Sample collected at the bottom of the tube	+	±	+	+	+	+
Air bubbles removed from the sample	±	±	+	+	+	+

Observations during the evaluation of short-term centrifugation in Kubik to facilitate air removal of the sample and collection of the sample. + = successful, ± partly successful. After a maximum of 2 min at 2 g all droplets of the sample had collected at the bottom of the tube. Gentle squeezing the tube at the center part where the clip had been improved the visual appearance of the sample.

**TABLE 4:** Test of mixing.

		1 min			2 min			2 min and squeezing		
Tube type	Valve end	V(S)	V(P)	V(S)/V(P)	V(S)	V(P)	V(S)/V(P)	V(S)	V(P)	V(S)/V(P)
C-Flex/RT2	Swabable	420	120	3.5	270	92	2.9	440	110	4
	Piercable	430	100	4.3	410	100	4.1	350	100	3.5
PVC/RT3	Swabable	440	120	3.7	350	110	3.2	220	70	3.1
	Piercable	320	110	2.9	150	30	5	370	80	4.6

Exemplary data of volumes (in µl) of supernatant V(S) and cell pellet V(P) recovered at the swabable and pierceable valve of C-Flex and PVC, after short-term centrifugation inside Kubik. V(S)/V(P): ratio of recovered volumes.

## 4 Discussion

The design and development of new hardware for blood tests suitable for inflight implementation is essential for monitoring astronaut health, conducting scientific experiments, and ensuring the success of space missions. We tested the feasibility of the new flight hardware design, the practicality of the developed crew procedure, and the biocompatibility of three tubing materials silicone, C-Flex, and PVC in a step-by-step approach. The hardware and the adapted CRA procedure proved to be eligible and precise in achieving the desired target volumes for the antigen mixture and the blood sample. Results from the parabolic flight campaign evidenced the feasibility of the procedure under microgravity conditions and revealed that the direct transfer of blood samples via a standard adapter was more efficient than indirect transfer. After the MBT, silicone was excluded. Due to the observed leaking mostly of PVC tubes during centrifugation speeds faster than 800 *g*, the procedure was adapted to a centrifugation speed of 600 *g*. Following the results from the various testing procedures, due to the potential risk of leakage for PVC, C-Flex was ultimately chosen as tubing material for the RTs. Storage stability of antigen solutions inside C-Flex tubes was proven for up to 9 months. Overall, the two-valve tube design was found easy to handle also during a parabolic flight. Centrifugation and clipping are feasible under microgravity and clipping results in a stable separation of supernatant and cells. The most critical aspect of the procedure is the presence of air inside the RT or the monovette. Air pockets inside the blood sampling tubes can have a critical impact on the transfer of blood into the RT and therefore the successful execution of the CRA. Air pockets inside the RT prevent the mixing of the two liquids. Careful handling of the monovette according to the procedure can help to prevent these problems.

New hardware is needed to gather further knowledge of the adaptation processes of the immune system. Unique challenges are encountered in Space when bio samples such as blood are processed mostly due to the physical laws of µg itself and the requirements for the safe handling of biological samples to protect astronauts from exposure to potential hazards. Further challenges are the need to interface with other necessary hardware onboard, here with Kubik. Creative and individual solutions are often the way forward since commercial tests and platforms have so far not been sustainably successful on board. These space-driven innovations can also have broader applications in healthcare and medical diagnostics and thus expand our competence on Earth. The CRA is a blood test that can be used to analyze proteins such as cytokines (i.e., cell-mediated immunity) but unlike a skin test, tissue-resident immune cells cannot be evaluated in blood (Samant et al., 2020). Thus, a combination of both a skin and a blood test would be ideal, indicating the need of development of a new skin test that is suitable for in-flight procedures.

Although the CRA might now be performed inflight and provide valuable data, the test still relies on the download of samples and analysis on ground. More frequent long-term missions in Low Earth Orbit and the upcoming Artemis missions will require a more elaborate infrastructure for health monitoring. Also due to the multistep procedure, the assay uses up a fair amount of crew time, which might be less favorable and more difficult to schedule. Although astronauts will become more and more accustomed to processing bio samples also for routine monitoring of health, automation of the incubation procedure could be a solution. Recently, devices for some standard parameters such as cortisol detection in saliva (Zangheri et al., 2019) or a white blood cell count and differential (Crucian et al., 2021) have been validated for use on board. These developments suggest that in the future, CRA samples might even be analyzed directly onboard in the form of a point-of-care method.

This hardware was validated with limited resources. A direct comparison between C-Flex/RT2 and PVC/RT3 did not show any significant differences between RT. This might at least in part be due to the low number of subjects and the way the test was planned. Unfortunately, a comprehensive testing campaign with a large number of subjects was not possible. Ideally, a ground study involving a control group similar in size to the astronaut cohort that will be studied might have revealed more differences between the RTs than we have seen. To prevent a potential bias by the hardware itself, the baseline data collections for the upcoming study will also be performed with the same hardware so that comparisons can be made and a ground testing campaign with C-Flex/RT2 alone is underway. Nevertheless, the test results presented here encourage confidence in the hardware design with C-Flex. This hardware might also provide a testing bed for the analysis of other specimens (cell cultures) or other types of assays and may therefore serve as a general testing platform. Successful commissioning of the hardware in the frame of the ESA project Immunity Assay was done in March 2023 (Figures 7D, E) and the first samples have already returned to the lab for analyses. A preliminary visual inspection of return samples suggests that the execution of the CRA on board has been successful.

## 5 Conclusion

The two-valve tubing design was found safe, stable, and feasible for use under microgravity conditions. The tubing material C-Flex passed the required tests and was found superior compared to two other materials and was therefore recommended for the intended application aboard the ISS. The multi-step procedure for the incubation of blood samples was successfully performed under microgravity during a parabolic flight experiment and further refined. The data are expected to provide a better insight into the adaptive changes of the human immune systems during long-term spaceflight in the frame of the ESA project Immunity Assay. The findings of this study hold important implications for space research and human spaceflight missions. The ability to monitor astronaut health in real-time, particularly during extended missions in space, is a critical advancement. Long-term space missions, including those to the Moon and Mars, require rigorous health monitoring to ensure the wellbeing of crew members. Real-time data on immune system responses and health parameters can inform decisions on crew activities, nutrition, and medical interventions, ultimately contributing to the success and safety of space exploration.

## Data Availability

The raw data supporting the conclusion of this article will be made available by the authors upon request, without undue reservation.
