# Supplementary material for: Monitoring functional immune responses with a cytokine release assay: ISS flight hardware design and experimental protocol for whole blood cultures executed under microgravity conditions
Source: Front Physiol. 2024 Jan 15;14:1322852. doi: 10.3389/fphys.2023.1322852 (PMC10823428; doi:10.3389/fphys.2023.1322852)
Supplement: Supplementary file 3 [file DataSheet1.docx]

Supplementary Material

**Monitoring functional immune responses with a Cytokine Release Assay: ISS Flight hardware design and experimental protocol for whole blood cultures executed under microgravity conditions**

**Judith-Irina Buchheim^1^, Matthias Feuerecker^1^, Michele Balsamo^2^, Marco Vukich^3,2^, Merel Van Walleghem^4,5^, Kevin Tabury^5^, Roel Quintens^5^, Randy Vermeesen^5^, Bjorn Baselet^5^, Sarah Baatout^5^, Bernd Rattenbacher^6^, Inês Antunes^3^, Thu Jennifer Ngo-Anh^3^, Brian Crucian^7^, Alexander Choukér^1^**^*^

^1^Laboratory of Translational Research “Stress and Immunity”, Department of Anesthesiology, LMU University Hospital, LMU Munich, Munich, Germany

^2^Kayser Italia S.r.l., Livorno, Italy

^3^European Space Research and Technology Centre (ESTEC), European Space Agency (ESA), Noordwijk, The Netherlands

^4^European Astronaut Center (EAC), European Space Agency (ESA), Cologne, Germany

^5^Belgian Nuclear Research Centre (SCK^.^CEN), Nuclear Medical Application Institute, Radiobiology Unit, Mol, Belgium

^6^Biotechnology Space Support Center (Biotesc), Lucerne University of Applied Sciences and Art (HSLU), Lucerne, Switzerland

^7^NASA Johnsons Space Center, Immunology Lab, Houston, TX, USA

*** Correspondence:** Prof. Dr. Alexander Choukér, [alexander.chouker@med.uni-muenchen.de](mailto:alexander.chouker@med.uni-muenchen.de)

# Supplementary Figure 1:

#
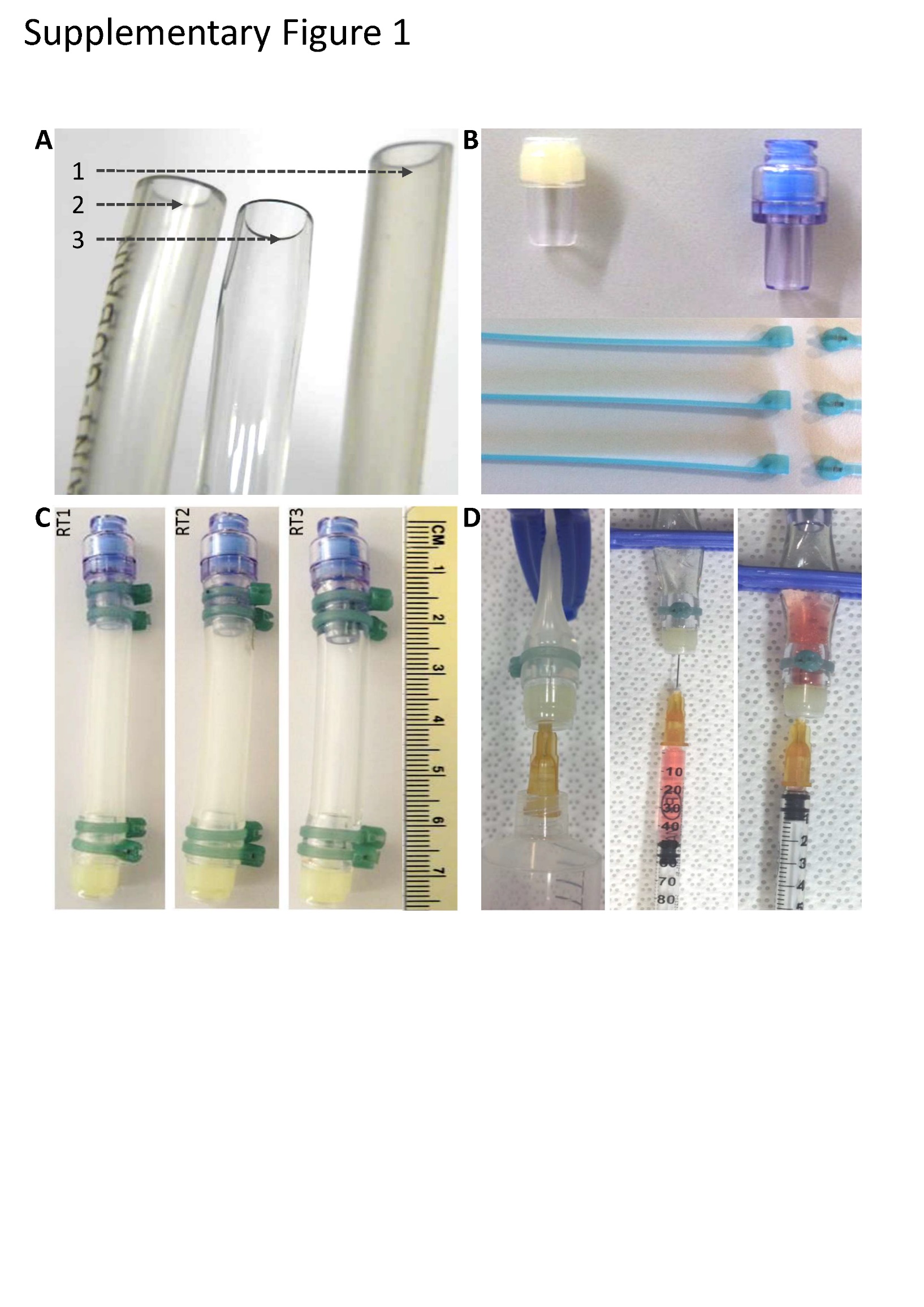


**Supplementary Figure 1 Reaction Tube design:** (A) Photo showing the different tubing materials evaluated in the study: silicone (1), C-Flex (2), and PVC (3). (B) Photos showing the yellow pierceable and the blue swabable valve (top) as well as the PTFE ties (bottom) used to secure the valve inside the tubing. (C) View on the assembled reaction tube (RT) design. RT1 (left) is made from silicone; RT2 (middle) from C-Flex and RT3 (right) from PVC. (D) Photos showing the steps necessary for prefilling of RTs. The clipped RT is evacuated on the side of the pierceable valve so that the compartment appears compressed (left). Next, the valve is pierced with a 26G cannula attached to a 1ml syringe containing 500µl of the desired antigen solution (middle). Vacuum-driven transfer of the solution is achieved effortlessly (right).

# Supplementary Figure 2:


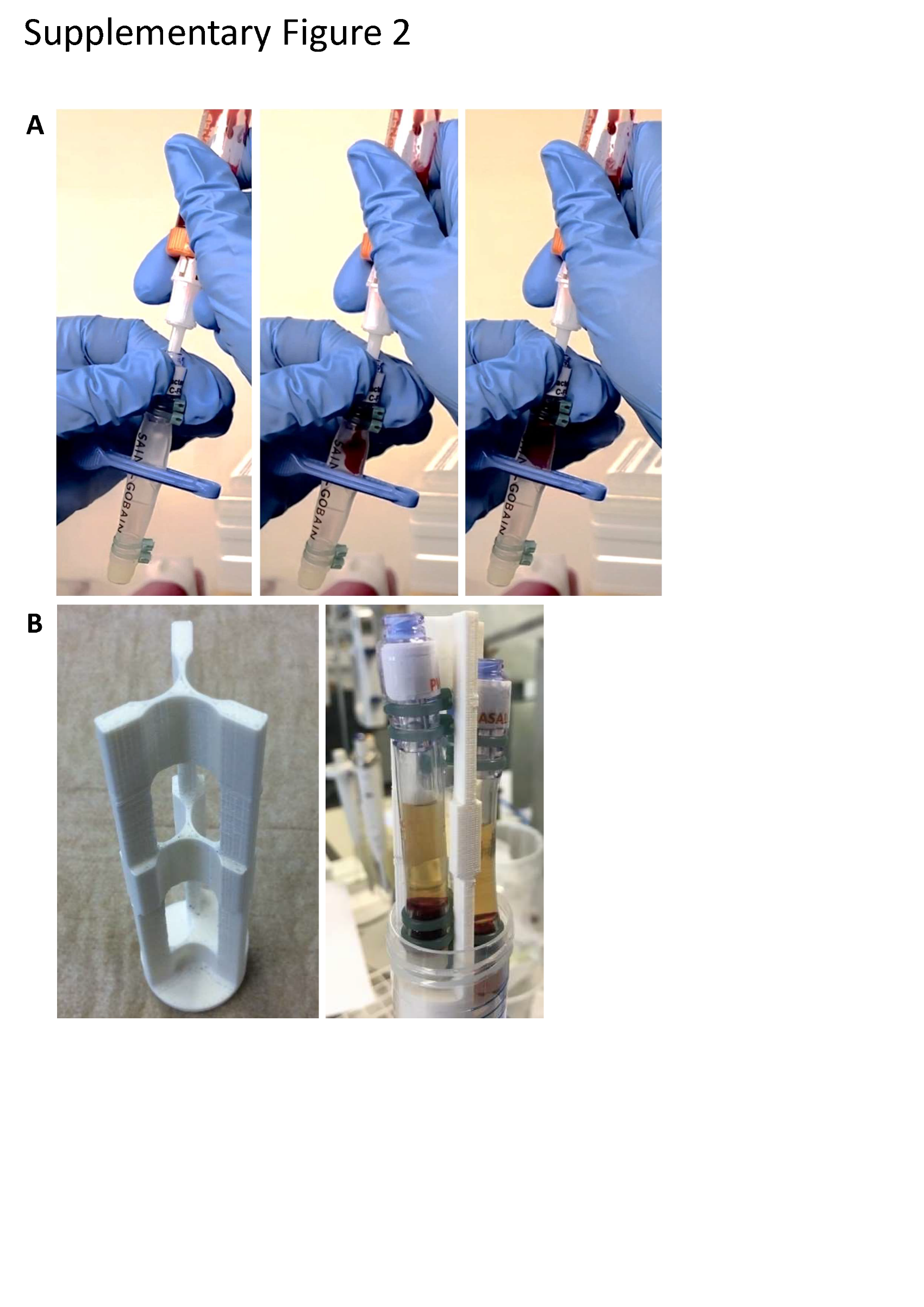


**Supplementary Figure 2.** (A) Exemplary sequential photos showing the successful transfer of the blood sample into an RT2/C-Flex type by connecting the blood sampling tube directly to the RT. (B) 3D-printed canonical tube insert designed for the centrifugation of RTs and to prevent twisting during centrifugation. (C) RTs directly after centrifugation show a clear separation of blood plasma and cells. Twisting did not occur.

# Supplementary Figure 3:


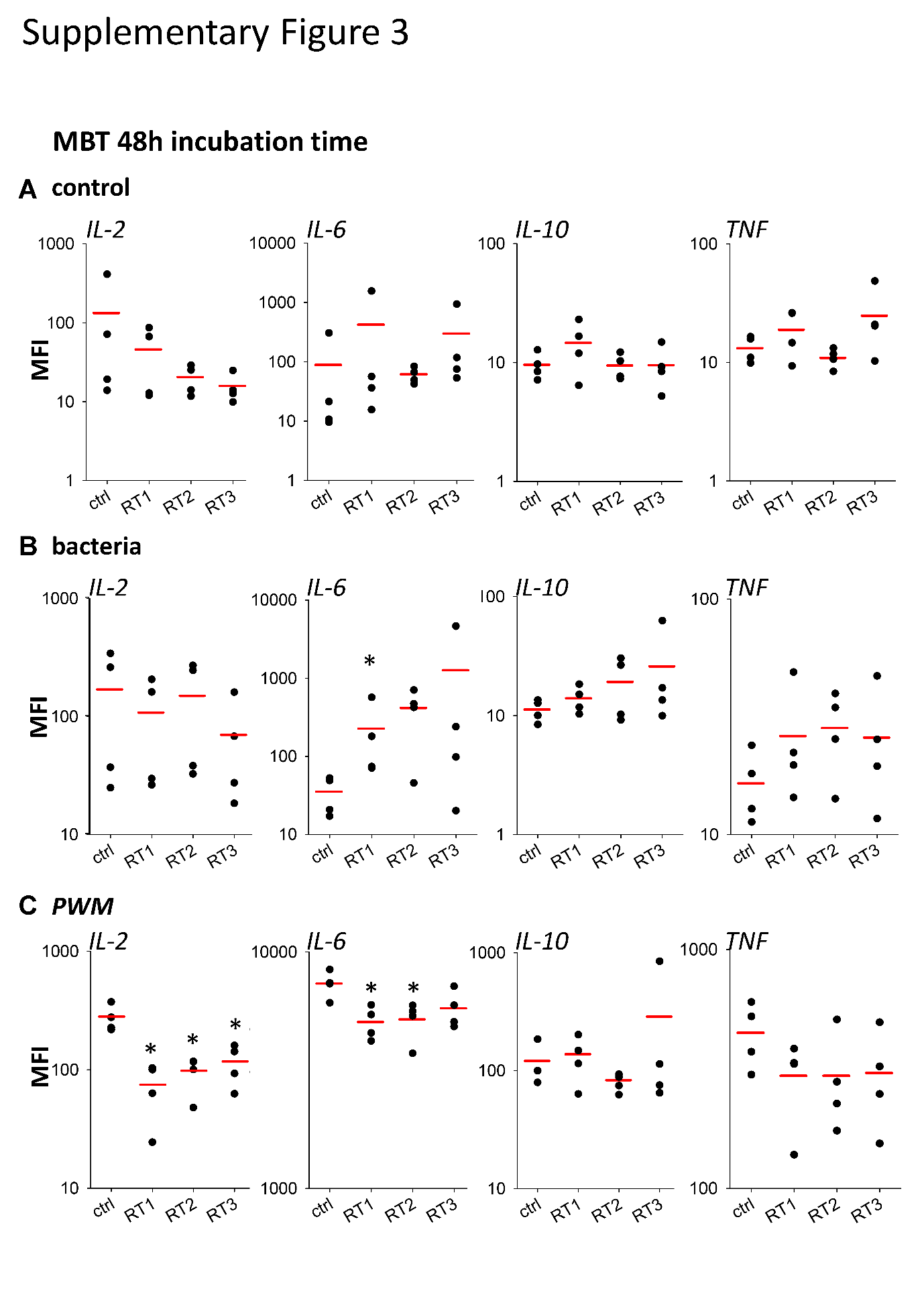


**Supplementary Figure 3 Biocompatibility of materials 48 hours:** Scatter plots showing amounts of IL-2, IL-6, IL-10, and TNF detected in supernatants after incubation of blood samples for 48 hours with RPMI (control) bacteria and PWM. The 3 types of RT Silicone (RT1), C-Flex (RT2), and PVC (RT3) were compared to the established standard setup (ctrl). The red line represents the mean, n=4 individual subjects, *P<0.05 vs. ctrl.

# Supplementary Table 1: MCT leakage test results for c-flex (C), PVC(P), and Silicone (S)

| RT | Pierceable valve | Swabable valve | Mass before test | Mass after test | m_loss_ | t | ρ | DP_1_ | DP_test_ | LR | Pass/ Fail |
| --- | --- | --- | --- | --- | --- | --- | --- | --- | --- | --- | --- |
| C-1 | PV-67 | SV-89 | 4,799 | 4,799 | 0,000 | 60 | 0,786 | 1 | 1 | 0,000 | PASS |
| C-2 | PV-68 | SV-90 | 4,778 | 4,778 | 0,000 | 60 | 0,786 | 1 | 1 | 0,000 | PASS |
| C-3 | PV-69 | SV-91 | 4,812 | 4,810 | 0,001 | 60 | 0,786 | 1 | 1 | 2,40E-07 | PASS |
| C-4 | PV-70 | SV-92 | 4,818 | 4,817 | 0,001 | 60 | 0,786 | 1 | 1 | 1,75E-07 | PASS |
| C-5 | PV-71 | SV-93 | 4,793 | 4,793 | 0,000 | 60 | 0,786 | 1 | 1 | 8,73E-08 | PASS |
| C-6 | PV-72 | SV-94 | 4,802 | 4,800 | 0,001 | 60 | 0,786 | 1 | 1 | 2,40E-07 | PASS |
| C-7 | PV-73 | SV-95 | 4,797 | 4,796 | 0,001 | 60 | 0,786 | 1 | 1 | 1,97E-07 | PASS |
| C-8 | PV-74 | SV-96 | 4,802 | 4,801 | 0,001 | 60 | 0,786 | 1 | 1 | 2,84E-07 | PASS |
| C-9 | PV-75 | SV-97 | 4,795 | 4,794 | 0,001 | 60 | 0,786 | 1 | 1 | 1,53E-07 | PASS |
| C-10 | PV-76 | SV-98 | 4,819 | 4,819 | 0,000 | 60 | 0,786 | 1 | 1 | 2,18E-08 | PASS |
| C-11 | PV-77 | SV-99 | 4,795 | 4,794 | 0,001 | 60 | 0,786 | 1 | 1 | 1,09E-07 | PASS |
| C-12 | PV-78 | SV-100 | 4,812 | 4,811 | 0,000 | 60 | 0,786 | 1 | 1 | 4,37E-08 | PASS |
| C-13 | PV-79 | SV-101 | 4,778 | 4,776 | 0,002 | 60 | 0,786 | 1 | 1 | 4,37E-07 | PASS |
| C-14 | PV-80 | SV-102 | 4,822 | 4,822 | 0,000 | 60 | 0,786 | 1 | 1 | 8,73E-08 | PASS |
| C-15 | PV-81 | SV-103 | 4,789 | 4,789 | 0,000 | 60 | 0,786 | 1 | 1 | 2,18E-08 | PASS |
| C-16 | PV-82 | SV-104 | 4,780 | 4,780 | 0,000 | 60 | 0,786 | 1 | 1 | 0,000 | PASS |
| C-17 | PV-83 | SV-105 | 4,975 | 4,974 | 0,001 | 60 | 0,786 | 1 | 1 | 3,06E-07 | PASS |
| C-18 | PV-84 | SV-106 | 4,793 | 4,792 | 0,001 | 60 | 0,786 | 1 | 1 | 1,97E-07 | PASS |
| C-19 | PV-85 | SV-107 | 4,802 | 4,801 | 0,001 | 60 | 0,786 | 1 | 1 | 1,31E-07 | PASS |
| C-20 | PV-86 | SV-108 | 4,874 | 4,873 | 0,001 | 60 | 0,786 | 1 | 1 | 2,40E-07 | PASS |
| C-21 | PV-87 | SV-109 | 4,849 | 4,849 | 0,000 | 60 | 0,786 | 1 | 1 | 8,73E-08 | PASS |
| C-22 | PV-88 | SV-110 | 4,908 | 4,908 | 0,001 | 60 | 0,786 | 1 | 1 | 1,09E-07 | PASS |
| P-23 | PV-67 | SV-89 | 5,492 | 5,490 | 0,002 | 60 | 0,786 | 1 | 1 | 3,71E-07 | PASS |
| P-24 | PV-68 | SV-90 | 5,334 | 5,334 | 0,000 | 60 | 0,786 | 1 | 1 | 6,55E-08 | PASS |
| P-25 | PV-69 | SV-91 | 5,360 | 5,354 | 0,007 | 60 | 0,786 | 1 | 1 | 1,44E-06 | PASS |
| P-26 | PV-70 | SV-92 | 5,444 | 5,441 | 0,003 | 60 | 0,786 | 1 | 1 | 5,68E-07 | PASS |
| P-27 | PV-71 | SV-93 | 5,503 | 5,502 | 0,001 | 60 | 0,786 | 1 | 1 | 2,40E-07 | PASS |
| P-28 | PV-72 | SV-94 | 5,432 | 5,423 | 0,008 | 60 | 0,786 | 1 | 1 | 1,81E-06 | PASS |
| P-29 | PV-73 | SV-95 | 5,398 | 5,388 | 0,010 | 60 | 0,786 | 1 | 1 | 2,18E-06 | PASS |
| P-30 | PV-74 | SV-96 | 5,448 | 5,447 | 0,001 | 60 | 0,786 | 1 | 1 | 1,31E-07 | PASS |
| P-31 | PV-75 | SV-97 | 6,286 | 6,285 | 0,001 | 60 | 0,786 | 1 | 1 | 2,84E-07 | PASS |
| P-32 | PV-76 | SV-98 | 5,520 | 5,519 | 0,001 | 60 | 0,786 | 1 | 1 | 1,97E-07 | PASS |
| P-33 | PV-77 | SV-99 | 6,299 | 6,298 | 0,001 | 60 | 0,786 | 1 | 1 | 1,75E-07 | PASS |
| P-34 | PV-78 | SV-100 | 5,544 | 5,542 | 0,001 | 60 | 0,786 | 1 | 1 | 3,06E-07 | PASS |
| P-35 | PV-79 | SV-101 | 6,310 | 6,310 | 0,001 | 60 | 0,786 | 1 | 1 | 1,53E-07 | PASS |
| P-36 | PV-80 | SV-102 | 5,272 | 5,268 | 0,005 | 60 | 0,786 | 1 | 1 | 9,83E-07 | PASS |
| P-37 | PV-81 | SV-103 | 5,512 | 5,511 | 0,001 | 60 | 0,786 | 1 | 1 | 3,06E-07 | PASS |
| P-38 | PV-82 | SV-104 | 6,339 | 6,339 | 0,000 | 60 | 0,786 | 1 | 1 | 6,55E-08 | PASS |
| P-39 | PV-83 | SV-105 | 5,526 | 5,524 | 0,002 | 60 | 0,786 | 1 | 1 | 4,80E-07 | PASS |
| P-40 | PV-84 | SV-106 | 5,606 | 5,605 | 0,001 | 60 | 0,786 | 1 | 1 | 2,18E-07 | PASS |
| P-41 | PV-85 | SV-107 | 6,202 | 6,201 | 0,002 | 60 | 0,786 | 1 | 1 | 3,28E-07 | PASS |
| P-42 | PV-86 | SV-108 | 5,465 | 5,455 | 0,010 | 60 | 0,786 | 1 | 1 | 2,27E-06 | PASS |
| P-43 | PV-87 | SV-109 | 5,485 | 5,484 | 0,001 | 60 | 0,786 | 1 | 1 | 1,53E-07 | PASS |
| P-44 | PV-88 | SV-110 | 6,314 | 6,314 | 0,000 | 60 | 0,786 | 1 | 1 | 4,37E-08 | PASS |
| S-45 | SV-89 | PV-67 | 5,107 | 5,104 | 0,003 | 60 | 0,786 | 1 | 1 | 6,55E-07 | PASS |
| S-46 | SV-90 | PV-68 | 5,151 | 5,148 | 0,003 | 60 | 0,786 | 1 | 1 | 6,77E-07 | PASS |
| S-47 | SV-91 | PV-69 | 5,113 | 5,110 | 0,003 | 60 | 0,786 | 1 | 1 | 6,33E-07 | PASS |
| S-48 | SV-92 | PV-70 | 5,133 | 5,130 | 0,003 | 60 | 0,786 | 1 | 1 | 6,77E-07 | PASS |
| S-49 | SV-93 | PV-71 | 5,084 | 5,081 | 0,003 | 60 | 0,786 | 1 | 1 | 5,46E-07 | PASS |
| S-50 | SV-94 | PV-72 | 5,326 | 5,323 | 0,003 | 60 | 0,786 | 1 | 1 | 5,89E-07 | PASS |
| S-51 | SV-95 | PV-73 | 5,125 | 5,122 | 0,003 | 60 | 0,786 | 1 | 1 | 7,21E-07 | PASS |
| S-52 | SV-96 | PV-74 | 5,269 | 5,266 | 0,003 | 60 | 0,786 | 1 | 1 | 7,21E-07 | PASS |
| S-53 | SV-97 | PV-75 | 5,128 | 5,125 | 0,003 | 60 | 0,786 | 1 | 1 | 6,99E-07 | PASS |
| S-54 | SV-98 | PV-76 | 5,152 | 5,149 | 0,003 | 60 | 0,786 | 1 | 1 | 6,55E-07 | PASS |
| S-55 | SV-99 | PV-77 | 5,131 | 5,128 | 0,003 | 60 | 0,786 | 1 | 1 | 6,33E-07 | PASS |
| S-56 | SV-100 | PV-78 | 4,873 | 4,870 | 0,003 | 60 | 0,786 | 1 | 1 | 7,20E-07 | PASS |
| S-57 | SV-101 | PV-79 | 5,234 | 5,231 | 0,003 | 60 | 0,786 | 1 | 1 | 7,21E-07 | PASS |
| S-58 | SV-102 | PV-80 | 5,141 | 5,138 | 0,003 | 60 | 0,786 | 1 | 1 | 6,55E-07 | PASS |
| S-59 | SV-103 | PV-81 | 5,187 | 5,183 | 0,003 | 60 | 0,786 | 1 | 1 | 6,77E-07 | PASS |
| S-60 | SV-104 | PV-82 | 5,096 | 5,093 | 0,003 | 60 | 0,786 | 1 | 1 | 6,55E-07 | PASS |
| S-61 | SV-105 | PV-83 | 5,156 | 5,153 | 0,003 | 60 | 0,786 | 1 | 1 | 6,11E-07 | PASS |
| S-62 | SV-106 | PV-84 | 5,125 | 5,122 | 0,003 | 60 | 0,786 | 1 | 1 | 6,33E-07 | PASS |
| S-63 | SV-107 | PV-85 | 5,112 | 5,109 | 0,003 | 60 | 0,786 | 1 | 1 | 7,42E-07 | PASS |
| S-64 | SV-108 | PV-86 | 5,242 | 5,239 | 0,003 | 60 | 0,786 | 1 | 1 | 6,99E-07 | PASS |
| S-65 | SV-109 | PV-87 | 5,144 | 5,141 | 0,003 | 60 | 0,786 | 1 | 1 | 6,77E-07 | PASS |
| S-66 | SV-110 | PV-88 | 5,178 | 5,175 | 0,003 | 60 | 0,786 | 1 | 1 | 7,20E-07 | PASS |

# Supplementary Video 1: The video shows the filling and unclipping of RT2 under microgravity conditions during the parabolic flight campaign.

# Supplementary Video 2: The video shows the filling and unclipping of RT3 under microgravity conditions during the parabolic flight campaign.
